# Mineral Element Contents in Commercially Valuable Fish Species in Spain

**DOI:** 10.1155/2014/949364

**Published:** 2014-04-23

**Authors:** Ana Rivas, Luis Peña-Rivas, Eduardo Ortega, Concepción López-Martínez, Fátima Olea-Serrano, Maria Luisa Lorenzo

**Affiliations:** ^1^Research Group on Nutrition, Diet and Risk Assessment (AGR255), Department of Nutrition and Food Science, Faculty of Pharmacy, University of Granada, Campus de Cartuja s/n, 18071 Granada, Spain; ^2^Granada Health Management Area, Andalusian Health Service, 18014 Granada, Spain; ^3^Department of Edaphology and Agricultural Chemistry, Faculty of Pharmacy, University of Granada, 18071 Granada, Spain; ^4^Department of Physical Chemistry, Faculty of Pharmacy, University of Granada, 18071 Granada, Spain

## Abstract

The aim of this study was to measure selected metal concentrations in *Trachurus trachurus*, *Trachurus picturatus*, and *Trachurus mediterraneus*, which are widely consumed in Spain. Principal component analysis suggested that the variable Cr was the main responsible variable for the identification of *T. trachurus*, the variables As and Sn for *T. mediterraneus*, and the rest of variables for *T. picturatus*. This well-defined discrimination between fish species provided by mineral element allows us to distinguish them on the basis of their metal content. Based on the samples collected, and recognizing the inferential limitation of the sample size of this study, the metal concentrations found are below the proposed limit values for human consumption. However, it should be taken into consideration that there are other dietary sources of these metals. In conclusion, metal contents in the fish species analyzed are acceptable for human consumption from a nutritional and toxicity point of view.

## 1. Introduction

Chromium (Chr), copper (Cu), iron (Fe), manganese (Mn), and zinc (Zn), among other metals, are essential micronutrients for humans [1] involved in important biological processes. These elements prevailingly play a functional and structural role in human body [2]. However, there are no homeostatic mechanisms in the human body to eliminate elements, such as cadmium (Cd), arsenic (As), mercury (Hg), and lead (Pb), which have no beneficial function for humans [3]. The toxicity of metals varies and is dependent on the concentrations at which a specific organism is exposed to them. However, long-term exposure to Hg, As, Cd, and Pb at relatively low levels can have deleterious effects on human health [4]. The European Food Safety Authority (EFSA) has provided evidence that exposure to these contaminants can cause neurological, cardiovascular and reproductive disorders [5].

Apart from occupational exposure to heavy metals, the population is primarily exposed to these metals via the diet [6]. For example, fish should be included in any healthy diet, as it is rich in proteins, low saturated fats, and omega fatty acids, which are beneficial for human health [7]. However, metals can concentrate in fish tissues, exceeding safety levels. Therefore, the consumption of fish involves exposure to some metals that may have a negative impact on human health [8–10]. Thus, human populations whose dietary habits include a high quantity of fish, seafood, and sea mammals are particularly exposed to metals. The Mediterranean diet is characterized by a high fish intake, especially in Spain. After Japan and Portugal, Spain is the third ranked country in fish consumption worldwide [5, 11].

Horse mackerel is one of the most consumed commercial fish in Spain. Three species are marketed under the name of horse mackerel: Atlantic horse mackerel (*Trachurus trachurus*), blue jack mackerel (*Trachurus picturatus*), and Mediterranean horse mackerel (*Trachurus mediterraneus*). All three belong to the family of Carangidae, which includes a variety of species with the anatomic characteristics typical of fish with high fat content [12]. In Spain, as in most countries, retailers and consumers do not make a clear distinction between these fish. The aim of this study was to measure selected metal concentrations in* Trachurus trachurus*,* Trachurus picturatus*, and* Trachurus mediterraneus*, which are widely consumed in Spain. The final goal of this study was to determine the correlation between metal concentrations in these fish species and human nutrition and health.

## 2. Material and Methods

### 2.1. Reagents

All reagents were of analytical reagent grade. Double deionized water was used for all dilutions. Nitric acid (65%), hydrogen peroxide (33%), vanadium pentoxide, and sodium borohydride were of suprapure quality (Merck, Darmstadt, Germany). All plasticware and glassware were cleaned by soaking in nitric acid (10%) and rinsed with double deionized water prior to use. Stock standard solutions, containing 1000 mgL^−1^ of metals (ICP multielement standard solution IV, Certipur, Titrisol, Merck) were prepared from standard vials. Standard reference material (National Research Council Canada, DORM-3) was used.

### 2.2. Apparatus

A Thermo Fisher/Unicam Solaar M5 (Waltham, MA, USA) atomic absorption spectrometer was used, which was fitted with Termo Fisher Scientific Unicam Mseries-Solaar M5 with GF95 graphite furnace and a VP90 Vapour System. Atomic absorption spectroscopy and atomic emission spectroscopy were performed to determine the presence of metals. The metals identified by atomic absorption spectroscopy were Ca, Mg, Zn (flame technique), Cu, Fe, Mn, Cr, Sn, Al, Cd, Pb (graphite furnace technique), As (hydride generation technique), and Hg (cold vapor technique). Atomic emission spectroscopy revealed the presence of Na and K (flame technique). For flame measurements, air/acetylene flame was used. For graphite furnace measurements, argon was used as inert gas. A deuterium arc lamp background corrector was used for the elements analyzed by atomic absorption spectroscopy with the graphite furnace technique and the hydride generation technique. The operating parameters for working elements are given in Tables 1 and 2. All determinations were done in triplicate.

### 2.3. Sampling

Between June and July 2010, samples were obtained from the following fish species:* Trachurus trachurus* (*n* = 48),* Trachurus mediterraneus* (*n* = 45), and* Trachurus picturatus* (*n* = 49). All samples included a sufficient number of individuals to minimize errors due to individual variations. Samples were purchased in local markets and large supermarket chains in Granada (Spain) and had not been necessarily caught in Granada coastal waters. They were randomly purchased independent of their geographical origins. Fish samples were packed in polyethylene bags and stored below −20°C until analysis.

### 2.4. Sample Preparation

A total of 200 g of edible tissue was filleted and dried in the microwave. Next, the samples were powdered using an agate mortar to prevent potential contamination of samples. Once the samples were powdered and homogenized, they were stored in polyethylene bottles at 5°C. Samples of 0.5 g were weighed in digestion vessels in triplicate adding 4 mL of HNO_3_ (Suprapur, Merck), 1 mL of H_2_O_2_ at 33%, and 5 mL of double deionized water. Once in the microwave vessels, digestion was performed as follows: maximum power 1000 w and initial temperature 20°C. After 20 minutes, the sample reached a temperature of 200°C, which was maintained for 20 minutes. Subsequently, the sample was cooled for a further 20 minutes. A blank digest was carried out in the same way. The accuracy of the method was checked by analyzed certified reference material.

### 2.5. Statistical Analysis

Data analyses were performed using the statistical package SPSS (version 11; SPSS, Chicago, IL). Metal concentrations were expressed as milligram and microgram per gram wet weight (ww). Values are given in means ± standards deviation (SD). Metal concentrations in different species were compared using one-way analysis of variance (ANOVA). Samples were considered significantly different at *P* < 0.05. Principal components analysis (PCA) was performed on data.

## 3. Results and Discussion

The accuracy and precision of analytical measurements were ensured using blank samples and fish protein certified reference material for trace metals. The results showed a high level of agreement between the certified and the analytical values (Table 3) and confirmed the suitability of our procedures and methodology.

Table 4 displays mean values for metal concentrations in edible muscles of the three* Trachurus* species. Although average concentrations of Al, Cr, and Ni did not differ significantly among species, there were statistically significant differences (*P* < 0.05) in metal concentrations among the species studied. Thus, the present study revealed that metal accumulation in fish is species specific. Toxic metal concentrations did not exceeded permissible limits in any of the samples.

Metals such as calcium, magnesium, sodium, and potassiumare essential since they play an important role in biological systems. The main role of these metals can be described as structural and functional. Structurally, they stand out for their role as integrators of organic compounds in the body. From a functional standpoint, they have a role in controlling important biological functions [13]. The concentrations of these metals in the fish samples used in the present study were comparable with the ranges reported by other authors [14, 15].* T. picturatus* was found to have significantly higher values of these metals as compared to the remaining two species (Table 4).

Lead is a ubiquitous toxic element, which is introduced into the environment by natural and anthropogenic processes. The natural contribution of lead comes from erosion of the Earth's crust and dust transport under varying climatic conditions, while anthropogenic sources of lead include emissions from industrial activities and the use of gasoline and phosphate fertilizers [16]. Cadmiumis a biotoxic element considered a priority pollutant and is widely employed in industrial products and processes. Anthropogenic inputs are considered the main source of Cd contamination in aquatic environments [16]. The levels of lead and cadmium found in this study are similar to those reported in previous studies on these fish species [2, 17, 18]. It should be pointed out that this study revealed that* T. picturatus* accumulate concentrations of lead (0.967 ± 0.135) and cadmium (0.321 ± 0.073) significantly higher than the other species studied.

Nickel concentrations are generally low in the aquatic environment [19]. The nickel concentrations in muscle tissue found in the present study were lower than reported values for* T. trachurus* (1.5 ± 0.13) from the Black Sea [18] and for* T. mediterraneus* (0.94 ± 0.76) from the Northeastern Mediterranean Sea in Turkey [20]. The muscles of* T. trachurus* had the highest nickel content, while those of* T. mediterraneus* had the lowest one.

Copper and zinc are considered essential elements for the fish organism, and their concentrations are regulated mainly by vital metabolic processes [16]. Copper is essential for human health but an overdose can cause adverse events such as liver and kidney damage [18, 21]. Zinc is known to be involved in most metabolic pathways in humans and deficient zinc levels can lead to loss of appetite, growth retardation, skin changes, and immunological abnormalities [18]. The values for zinc and copper reported in this study are in agreement with those reported for* T. trachurus* [18, 22] and* T. mediterraneus* species [17] in previous studies. This study showed that* T. picturatus* contained copper and zinc levels two orders of magnitude lower than the other two fish species studied.

Arsenic is a widely distributed contaminant that occurs both naturally and as result of human activity [23]. Arsenic levels in the fish samples analyzed were lower than those reported in the literature available on these fish species [2, 18]. The results obtained in this study should be taken into consideration, as arsenic is known to be carcinogenic in humans, causing lung, liver, skin, and bladder cancer [24]. The lower levels of arsenic in these fish make them ideal for consumption, especially for particularly vulnerable groups of people.

Iron is found in organism compounds such as hemoglobin or myoglobin, as well as ferritin and hemosiderin in fish liver. The mean Fe contents found in this study ranged from 4.48 to 14.52 *μ*g/g, which is lower than the values reported for* T. mediterraneus* in the Iskenderun Bay (41.84 ± 18.41 *μ*g/g) and for* T. trachurus* in Turkey (145 ± 12 *μ*g/g). However, these results are in agreement with the values reported in Spanish and USDA food composition databases.

Chromium and manganese are recognized as essential trace elements for humans, of which several metabolic roles have been determined. These include manganese-containing enzymes and chromium involved in insulin function [2]. The lowest and highest chromium levels found were 0.092 *μ*g/g for* T. mediterraneus* and 0.126 *μ*g/g for* T. trachurus*. The minimum and maximum manganese levels observed were 0.088 *μ*g/g for* T. trachurus* and 0.168 *μ*g/g for* T. mediterraneus*. Chromium and manganese concentration levels were lower than those reported by Mendil et al. [2] for these species in Turkey.

Aluminum occurs naturally in the environment and is also released as a result of anthropogenic activities such as mining and industrial activity and in the production of aluminium metal and other aluminium compounds. The primary source of aluminium exposure for the general population is through food. Tin has not been proven to be nutritionally essential for humans and exposure to high levels of tin can cause gastrointestinal irritation and upset [25]. The highest values of aluminium and tin were found in* T. mediterraneus* with a concentration of 5.813 and 0.057 *μ*g/g, respectively. These values, when compared with the provisional tolerable weekly intake (PTWI) of 1 mg/Kg of body weight (bw) for aluminium [25] and 14 mg/Kg bw for tin [26], indicate that aluminium contents in the edible parts of these fish species does not represent a significant portion of human daily intake.

Mercury is known to be a human toxicant. The primary source of contamination for humans is fish [18]. Mercury concentrations ranged from 0.146 ± 0.097 for* T. trachurus* to 0.286 ± 0.05 for* T. picturatus*, which is in agreement with the data reported by Roméo et al. [22] for* T. trachurus* from the Mauritanian Coast. Perelló et al. [1] also found similar mercury levels in Spanish fish. The Hg concentrations found in analyzed fishes are below the proposed limit set by European regulation.

**Figure 1:**
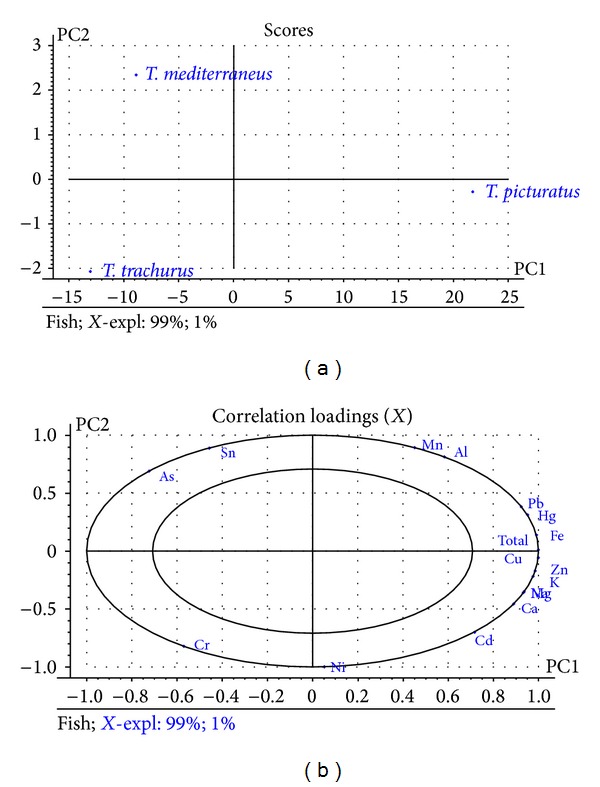
Score plot (a) and loading plot (b) of principal component analysis applied to the data set.

Principal component analysis was performed to explore data distribution patterns and visualize the potential relationship between the studied metals and fish species (see Figure 1). The first dimension PC-1 (which accounted for 99% of total variance) allowed us to distinguish fish species. Comparisons between the PCA plots suggested that the variable Cr was the main responsible variable for the identification of* T. trachurus*, the variables As and Sn for* T. mediterraneus*, and the rest of variables for* T. picturatus*. This well-defined discrimination between fish species provided by mineral element allows us to distinguish them on the basis of their metal content.

Concentrations of the toxic metals Pb, Cd, As, and Hg in human body due to fish intake were assessed and are displayed in Table 5. According to the Spanish Agency for Food Safety (AESAN), the daily consumption of horse mackerel in the Spanish adult population is 0.49 g per day. The weekly intake of these metals per Kg of body weight was calculated taking 68.48 Kg as the average weight of the Spanish population [27]. Subsequently, weekly intake values were compared with the provisional tolerable weekly intake (PTWI) values established by the World Health Organization standards. The TWI values for the metals studied were below standard values. Compared to the PTWI proposed by the WHO, the dietary intake of lead and arsenic were low; this is in agreement with the very low pollution levels reported in numerous studies on the Mediterranean countries and other areas [5, 28]. However, cadmium and mercury intake levels were comparatively high. The minimum mean weekly intake of these metals in the population amounted to 0.28% of the PTWI for cadmium and 0.45% for mercury, while the maximum amounted to 0.64% for cadmium and 0.89% for mercury.

## 4. Conclusion

In this study, we determined metal concentrations in muscle tissue of three popular commercial species in Spain and assessed if the intake of these species might represent a health risk. This is the first study to analyze metal concentrations in* Trachurus trachurus*,* Trachurus picturatus*, and* Trachurus mediterraneus *spp., all found in the Spanish market, and which, to the best of our knowledge, have not been previously studied. Based on the samples collected, and recognizing the inferential limitation of the sample size of this study, the metal concentrations found are below the proposed limit values for human consumption. Although mean Al, Cr, and Ni concentrations were similar among the species studied, the concentrations of other metals differed. Principal component analysis suggested that the variable Cr was the main responsible variable for the identification of* T. trachurus*, the variables As and Sn for* T. mediterraneus*, and the rest of variables for* T. picturatus*. This well-defined discrimination between fish species provided by mineral element allows us to distinguish them on the basis of their metal content. These results can be used to understand the nutritional quality of these fish species and evaluate the potential risks associated with their consumption. In conclusion, metal contents in the fish species analyzed are acceptable for human consumption from a nutritional and toxicity point of view. However, it should be taken into consideration that there are other dietary sources for these metals.

## Figures and Tables

**Table 1:** Instrumental analytical conditions for atomic absorption spectrometry (flame and hydride generation technique) and for atomic emission spectrometry (flame technique) of investigated elements.

	Wavelength (nm)	Slit width (nm)	Flow air/acetylene (L/min)	Lamp current (%)
FAAS				
Ca	422.7	0.5	1.2	100
Mg	285.2	0.5	0.9	75
Zn	213.9	0.5	0.9	75
FAES				
Na	589.0	0.5	1.1	0
K	766.5	0.5	1.1	0

	Wavelength (nm)	Slit width (nm)	Flow argon (L/min)	Lamp current (%)

HGAAS				
As	193.7	1	0.2	75
CVAAS				
Hg	253.7	0.5	0.2	75

FAAS: flame atomic absorption spectrometry; FAES: flame atomic emission spectrometry; HGAAS: hydride generation atomic absorption spectrometry; CVAA: cold vapour atomic spectrometry.

**Table 2:** Instrumental analytical conditions for graphite furnace atomic spectrometry of investigated elements.

	Cu	Fe	Mn	Cr	Sn	Al	Cd	Pb	Ni
Wavelength (nm)	324.8	248.3	279.5	357.9	224.6	309.3	228.8	217.0	232.0
Slit width (nm)	0.5	0.2	0.2	0.5	0.5	0.5	0.5	0.5	0.1
Lamp current (%)	80	100	80	100	80	80	50	90	80
Sample volume (uL)	20	20	20	20	20	20	20	20	20
Matrix modifier/volume (uL)	—	Mg(NO_3_)_2_/0.5	—	—	Mg(NO_3_)_2_/0.5	—	Pd(NO_3_)_2_/0.5	NH_4_H_2_PO_4_/0.5	Mg(NO_3_)_2_/0.5
Argon flow (mL/min)	0.2	0.2	0.2	0.2	0.2	0.2	0.2	0.2	0.2
Drying^a^	150/40/10	150/30/10	150/30/10	120/40/12	100/30/10	120/30/5 150/20/15	120/30/10	120/30/10	150/30/10
Ashing^a^	400/20/10	450/20/10	900/20/150	750/20/630	800/20/150	800/20/5	450/20/150	400/10/30	450/20/10
Pre-atomization^a^	850/20/150	1100/20/150	—	1200/30/450	—	1500/15/20	750/20/150	800/20/150	1000/20/150
Atomization^a^	2100/3/0	2100/3/0	2100/3/0	2500/3/0	2300/3/0	2450/3/0	1500/3/0	1450/3/0	2500/3/0
Cleaning^a^	2700/3/0	2700/3/0	2700/3/0	2700/3/0	2700/3/0	2700/3/0	2700/3/0	2700/3/0	2700/3/0

^a^Temperature (°C)/time (s)/ramp (°C/s).

**Table 3:** Reference material recovery study.

Element	Certified value (*μ*g g^−1^)	Obtained value (*μ*g g^−1^)	Recovery (%)	RSD (%)
Fe	347 ± 20	343 ± 18	98.8	5.24
Zn	51.3 ± 3.1	53.21 ± 3.48	103.7	6.54
Hg	0.382 ± 0.06	0.370 ± 0.02	97.0	5.40
As	6.88 ± 0.30	6.811 ± 0.46	99.0	6.75
Cr	1.89 ± 0.17	1.92 ± 0.12	101.5	6.25
Cu	15.50 ± 0.63	15.11 ± 0.41	97.5	2.71
Ni	1.28 ± 0.24	1.25 ± 0.13	98.3	10.4
Pb	0.395 ± 0.05	0.386 ± 0.02	97.8	5.18
Cd	0.290 ± 0.02	0.284 ± 0.01	98.0	3.52
Sn	0.066 ± 0.012	0.064 ± 0.002	97.3	3.12

Mean ± standard deviation; RSD: relative standard deviation.

**Table 4:** Concentration of metals (mg g^−1^ and *μ*g g^−1^ wet weight) in fish species studied average concentrations ± standard deviation.

Element	Total	*T. trachurus *	*T. mediterraneus *	*T. picturatus *	One-way *F* value
Ca (mg g^−1^)	1.1533 ± 0.430	1.137 ± 0.063	0.879 ± 0.346	1.594 ± 0.785	14.84*
Mg (mg g^−1^)	0.325 ± 0.134	0.305 ± 0.066	0.241 ± 0.038	0.489 ± 0.226	23.73*
Na (mg g^−1^)	1.481 ± 1.477	1.282 ± 0.647	0.602 ± 0.075	3.195 ± 2.798	19.27*
K (mg g^−1^)	3.570 ± 0.994	3.314 ± 0.273	3.117 ± 0.626	4.761 ± 1.786	18.56*
Cu (*μ*g g^−1^)	0.819 ± 0.724	0.509 ± 0.190	0.586 ± 0.220	1.788 ± 1.253	28.66*
Fe (*μ*g g^−1^)	7.575 ± 5.225	4.485 ± 0.749	7.064 ± 6.028	14.523 ± 2.928	34.31*
Mn (*μ*g/g)	0.129 ± 0.081	0.088 ± 0.384	0.168 ± 0.107	0.154 ± 0.114	6.364*
Zn (*μ*g g^−1^)	13.855 ± 8.399	11.518 ± 1.376	10.656 ± 3.419	23.327 ± 17.416	14.65*
Cr (*μ*g g^−1^)	0.108 ± 0.052	0.126 ± 0.045	0.092 ± 0.053	0.093 ± 0.089	2.664
Sn (*μ*g g^−1^)	0.047 ± 0.015	0.043 ± 0.013	0.057 ± 0.019	0.042 ± 0.007	5.749*
Al (*μ*g g^−1^)	4.314 ± 5.317	2.408 ± 1.600	5.857 ± 8.357	5.813 ± 7.260	2.748
As (*μ*g g^−1^)	0.034 ± 0.016	0.032 ± 0.014	0.043 ± 0.023	0.025 ± 0.0002	5.444
Cd (*μ*g g^−1^)	0.238 ± 0.042	0.253 ± 0.056	0.141 ± 0.042	0.321 ± 0.073	10.84*
Hg (*μ*g g^−1^)	0.212 ± 0.914	0.146 ± 0.097	0.204 ± 0.043	0.286 ± 0.05	10.09*
Pb (*μ*g g^−1^)	0.817 ± 0.125	0.672 ± 0.143	0.814 ± 0.120	0.967 ± 0.135	10.83*
Ni (*μ*g g^−1^)	0.150 ± 0.153	0.193 ± 0.200	0.087 ± 0.066	0.155 ± 0.202	2.386

*P < 0.05.

**Table 5:** Accumulation level of metals in humans due to consumption of studied fish week^−1^Kg^−1^ body wt.

Element	Total	*T. trachurus *	*T. mediterraneus *	*T. picturatus *
Pb (*μ*g kg^−1^)				
PTWI_std _	25	25	25	25
TWI_cal_	0.0409	0.0336	0.0407	0.0484
(TWI_cal _/PTWI_std_) × 100	0.1636	0.1346	0.1630	0.1937
Cd (*μ*g kg^−1^)				
PTWI_std _	2.5	2.5	2.5	2.5
TWI_cal_	0.0119	0.0126	0.0070	0.0160
(TWI_cal _/PTWI_std_) × 100	0.47	0.5068	0.2824	0.643
As (*μ*g kg^−1^)				
PTWI_std _	15	15	15	15
TWI_cal_	0.001702	0.001602	0.00215	0.001252
(TWI_cal _/PTWI_std_) × 100	0.0113	0.01068	0.0413	0.00834
Hg (*μ*g kg^−1^)				
PTWI_std _	1.6	1.6	1.6	1.6
TWI_cal_	0.01061	0.0073	0.0102	0.014
(TWI_cal _/PTWI_std_) × 100	0.663	0.457	0.638	0.895

PTWI_std_: standard provisional tolerable weekly intakes; TWI_cal_: calculated tolerable weekly intakes.
